# The alarming outbreaks of dengue in Nepal

**DOI:** 10.1186/s41182-020-0194-1

**Published:** 2020-02-07

**Authors:** Niran Adhikari, Dinesh Subedi

**Affiliations:** 1 Animal Health Training & Consultancy Services, AHTCS, Pokhara, Nepal; 2 School of Biological Sciences, Monash University, Melbourne, Australia

**Keywords:** Dengue, Nepal, Alarming outbreaks, Control, Visit Nepal Year 2020

## Abstract

Dengue is a mosquito-borne viral infection. Since the first reported incidence in 2004, several sporadic outbreaks of dengue have been recorded from both tropical and subtropical regions of Nepal, including the capital city Kathmandu. However, in the last 5 years, the incidence of dengue cases has risen alarmingly. The largest-ever outbreak was reported in 2019, which killed six people. The global warming, unplanned urbanization, increased transportation, and lack of efficient mosquito control are presumably associated with the spread of dengue and its vector to the plane and hilly regions of this country. With the ongoing Nepalese government campaign “Visit Nepal Year 2020” to attract two million tourists in mind, effective dengue control measures must be implemented to control potential future outbreaks.

Dear Editor,

Nepal is a landlocked country located between India and China. Several studies have shown that dengue is well established in neighboring countries and the incidence of dengue is in the rise in both India and China [1, 2]. Given the open border policy with India and a tremendous trade relationship with China, Nepal is always in a high risk of cross-country spread of dengue.

Dengue is caused by the dengue virus (DENV) of family *Flaviviridae*. There are four well-established serotypes of dengue virus (DENV1–4) that infect humans. It is mainly transmitted by *Aedes aegypti* and *Aedes albopictus* female mosquitoes [3]. The first reported incidence of dengue in 2004 was considered to be imported to Nepal from India, based on genetic similarity [4, 5]. Shortly after the first incidence, several cases of dengue have been reported from tropical lowland and subtropical hilly region, including Kathmandu and surrounding cities. In 2006, all four serotypes of the dengue virus were reported [6]. The dengue outbreaks have gradually stretched out towards the hills and mountains from plane tropical regions [7].

The major clinical symptoms of dengue infection in Nepalese patient reported by Khetan et al. were fever (100%), cerebral pain (71.3%), rashes (11.3%), retro-orbital torment (23.5%), retching (23.4%), joint pain (32.1%), and thrombocytopenia (85.7%) [8]. During the first decade (2004–2013) of Nepalese dengue history, two major epidemics in 2010 and 2013 have been recorded [9]. However, in the last 5 years (2014–2019), a yearly outbreak of the dengue has been more frequent compared with 2004–2013. The number of confirmed annual dengue incidence varied from 336 to 14,662 between 2014 and 2019 (Fig. 1) [10]. The largest-ever outbreak was reported in 2019, infecting more than 14,000 people. Similar to previous years, the outbreak of 2019 has started in midsummer from a tropical region and then spread to hilly subtropical location [11]. In the year 2019, the first dengue case was confirmed in Sunsari District, eastern region of Nepal, on May 13, thereafter, in Makwanpur District, southwest district of Kathmandu, on July 27, and in next 2 months, dengue covered 68 out of 77 districts in Nepal [12]. A total of six deaths have been reported in 2019 [13]. The regional distribution of 2019 dengue outbreak is shown in Fig. 2. The highest number of dengue infection (7151) was noted in Province 3 (Bagmati), which includes the capital, Kathmandu. There were 1583 confirmed dengue cases in Kathmandu alone [13]. Although the major cause of dengue outburst in Kathmandu, which is otherwise considered as not suitable for dengue vectors, is largely unknown, unplanned housing, improper waste management, and highly dense population could be the factors that help the spread of dengue mosquitoes. This alarming situation of the dengue outbreak in Nepal has attracted the attention of international media [14]. This may influence the campaign of “Visit Nepal Year 2020” where it targets two million tourists [15], whose main route of entry is Kathmandu. Accordingly, such outbreaks do not just influence the national economy, but also expands the dengue hazard to other nations. Given that the majority of the Nepalese population resides in tropical and subtropical regions, more than 50% of people in Nepal are in the risk of dengue infection [7].

**Fig. 1:**
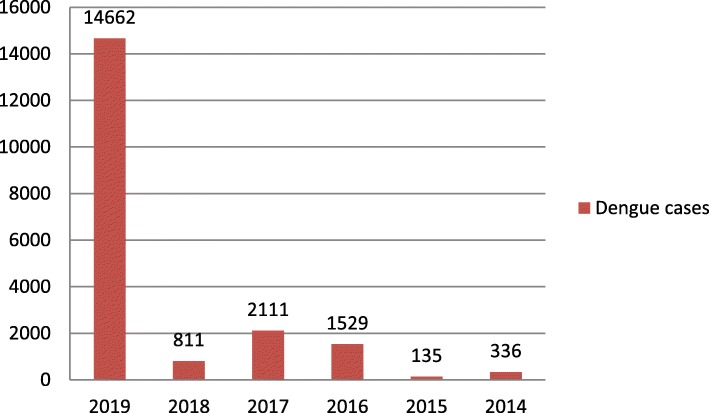
Year-wise records of dengue cases in Nepal from 2013/2014–2018/2019. Data were taken from the Epidemiology Disease Control Division, Nepal, which records confirmed cases of dengue

**Fig. 2:**
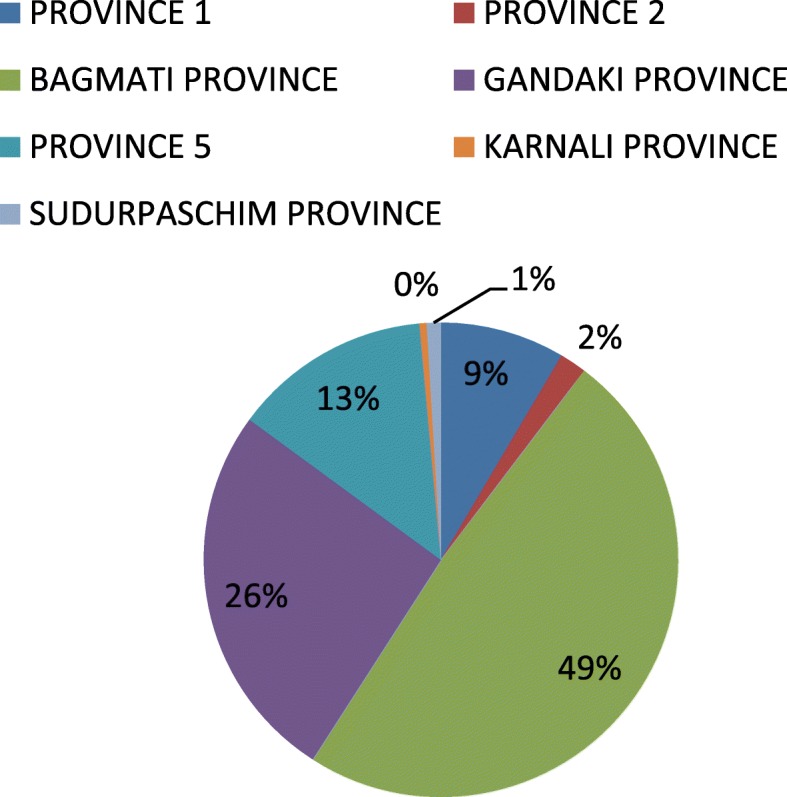
Region-wise records of dengue cases in Nepal, 2019. Data were taken from the Epidemiology Disease Control Division, Nepal, which records confirmed cases of dengue

The present situation has indicated that the dengue infections could be explosive in future in this country and currently available disease control measures are not effective to control the transmission of dengue. The government has released national guidelines for the prevention, control, and management of dengue in Nepal [16]. The vector control method is one of the proven control methodologies. There are practices of using insecticide-treated mosquito bed nets in Nepal to control other mosquito-borne infections such as malaria and Kala-zar. However, dengue vectors are diurnal, and this practice does not seem to be protective for dengue [16]. Therefore, there is a need for effective vector surveillance program and awareness to the general public on strategies to control transmission and proliferation of dengue vector. With the increasing trend of visiting overseas in Nepalese community, the risk of dengue transmission from countries other than India and China should also be investigated. Furthermore, the case reported by “Epidemiology Disease Control Division, Nepal” needs to be verified by molecular techniques such as polymerase chain reaction (PCR). This should be supported by well-equipped virology laboratories to track the transmission of dengue serotypes.

## Data Availability

The datasets generated and/or analyzed during the current study are available in the EDCD|Dengue Control Program [Internet] [cited 2019 Nov. 8]. Available from http://www.edcd.gov.np/section/dengue-control-program

## Abbreviations

DENV: Dengue virus
PCR: Polymerase chain reaction
